# The Changing Landscape of Respiratory Viruses Contributing to Hospitalizations in Quebec, Canada: Results From an Active Hospital-Based Surveillance Study

**DOI:** 10.2196/40792

**Published:** 2024-05-06

**Authors:** Rodica Gilca, Rachid Amini, Sara Carazo, Radhouene Doggui, Charles Frenette, Guy Boivin, Hugues Charest, Jeannot Dumaresq

**Affiliations:** 1 Direction des risques biologiques Institut national de santé publique du Québec Québec, QC Canada; 2 Research Center of Centre hospitalier universitaire de Québec-Université Laval Québec, QC Canada; 3 Département de médecine préventive Université Laval Québec, QC Canada; 4 Department of Medicine Division of Infectious Diseases McGill University Health Center Montreal, QC Canada; 5 Laboratoire de santé publique Institut national de santé publique du Québec Montreal, QC Canada; 6 Departement of Microbiology and Infectiology Centre intégré de santé et de services sociaux de Chaudière-Appalaches Levis, QC Canada

**Keywords:** respiratory viruses, SARS-CoV-2, COVID-19, hospitalizations, acute respiratory infections, children, adults, coinfections, prepandemic, pandemic

## Abstract

**Background:**

A comprehensive description of the combined effect of SARS-CoV-2 and respiratory viruses other than SARS-CoV-2 (ORVs) on acute respiratory infection (ARI) hospitalizations is lacking.

**Objective:**

This study aimed to compare the viral etiology of ARI hospitalizations before the pandemic (8 prepandemic influenza seasons, 2012-13 to 2019-20) and during 3 pandemic years (periods of increased SARS-CoV-2 and ORV circulation in 2020-21, 2021-22, and 2022-23) from an active hospital-based surveillance network in Quebec, Canada.

**Methods:**

We compared the detection of ORVs and SARS-CoV-2 during 3 pandemic years to that in 8 prepandemic influenza seasons among patients hospitalized with ARI who were tested systematically by the same multiplex polymerase chain reaction (PCR) assay during periods of intense respiratory virus (RV) circulation. The proportions of infections between prepandemic and pandemic years were compared by using appropriate statistical tests.

**Results:**

During prepandemic influenza seasons, overall RV detection was 92.7% (1384/1493) (respiratory syncytial virus [RSV]: 721/1493, 48.3%; coinfections: 456/1493, 30.5%) in children (<18 years) and 62.8% (2723/4339) (influenza: 1742/4339, 40.1%; coinfections: 264/4339, 6.1%) in adults. Overall RV detection in children was lower during pandemic years but increased from 58.6% (17/29) in 2020-21 (all ORVs; coinfections: 7/29, 24.1%) to 90.3% (308/341) in 2021-22 (ORVs: 278/341, 82%; SARS-CoV-2: 30/341, 8.8%; coinfections: 110/341, 32.3%) and 88.9% (361/406) in 2022-23 (ORVs: 339/406, 84%; SARS-CoV-2: 22/406, 5.4%; coinfections: 128/406, 31.5%). In adults, overall RV detection was also lower during pandemic years but increased from 43.7% (333/762) in 2020-21 (ORVs: 26/762, 3.4%; SARS-CoV-2: 307/762, 40.3%; coinfections: 7/762, 0.9%) to 57.8% (731/1265) in 2021-22 (ORVs: 179/1265, 14.2%; SARS-CoV-2: 552/1265, 43.6%; coinfections: 42/1265, 3.3%) and 50.1% (746/1488) in 2022-23 (ORVs: 409/1488, 27.5%; SARS-CoV-2: 337/1488, 22.6%; coinfections: 36/1488, 2.4%). No influenza or RSV was detected in 2020-21; however, their detection increased in the 2 subsequent years but did not reach prepandemic levels. Compared to the prepandemic period, the peaks of RSV hospitalization shifted in 2021-22 (16 weeks earlier) and 2022-23 (15 weeks earlier). Moreover, the peaks of influenza hospitalization shifted in 2021-22 (17 weeks later) and 2022-23 (4 weeks earlier). Age distribution was different compared to the prepandemic period, especially during the first pandemic year.

**Conclusions:**

Significant shifts in viral etiology, seasonality, and age distribution of ARI hospitalizations occurred during the 3 pandemic years. Changes in age distribution observed in our study may reflect modifications in the landscape of circulating RVs and their contribution to ARI hospitalizations. During the pandemic period, SARS-CoV-2 had a low contribution to pediatric ARI hospitalizations, while it was the main contributor to adult ARI hospitalizations during the first 2 seasons and dropped below ORVs during the third pandemic season. Evolving RVs epidemiology underscores the need for increased scrutiny of ARI hospitalization etiology to inform tailored public health recommendations.

## Introduction

Stringent mitigation efforts, such as border closures, travel restrictions, lockdowns, social distancing, use of masks in public spaces, school and business closures, and remote work, had been implemented worldwide to reduce the transmission of SARS-CoV-2 and its impact on hospital bed capacity [1]. While the first weeks of 2020 in the Northern Hemisphere were dominated by respiratory viruses other than SARS-CoV-2 (ORVs), SARS-CoV-2 almost completely replaced seasonally circulating ORVs within several weeks [2-4]. Public health measures in response to the pandemic altered traditional seasonality of some respiratory viruses (RVs), with virtual disappearance of others during extended periods of time in different parts of the world [5-10]. Among children, mitigation efforts led to a decrease in pediatric visits and hospitalizations overall and especially in those having acute respiratory infections (ARIs) [11,12], bronchiolitis [13,14], and pediatric asthma exacerbations associated with ARIs [15]. Easing of public health measures was accompanied by a subsequent surge of ORV circulation [16-18] and gradual return to usual seasonal patterns [19,20]. An interseasonal surge in RSV hospitalizations occurred in 2021 in winter instead of the usual summer season in Australia [16] and during the summer-fall months instead of winter in Canada [20]. In 2022, an unusually late influenza season was observed in the Northern Hemisphere [20] and an earlier than usual RSV hospitalization peak occurred between October and December [19,21].

The pandemic had an impact on laboratory resources, with lower volumes of performed tests and changes in the propensity to test for ORVs, especially during the first months, as well as on health-seeking behaviors, which may complicate the interpretation of ORV surveillance. Detection of ORVs in hospitalized patients may be more informative since admission requires a certain degree of severity and the propensity to be tested for a larger panel of RVs is higher. However, because of the high demand of SARS-CoV-2 tests in hospital laboratories, testing for ORVs was reduced even in hospitalized patients during the first stages of the pandemic.

A number of reports described detection of ORVs in patients hospitalized with ARI or COVID-19 during the pandemic [9,22-26]. However, to our knowledge, no report has described the results of the systematic detection of both ORVs and SARS-CoV-2 (not only at the physician request) using a panel of multiple RVs in a multicenter network including both pandemic years and comparing them with as long as 8 prepandemic years. The characterization of the combined impact of both SARS-CoV-2 and ORVs on ARI hospitalizations during the 3 pandemic years and its comparison with prepandemic seasons may provide insightful information on the postpandemic period when SARS-CoV-2 is expected to cocirculate along with ORVs.

In Quebec, Canada, a prospective hospital-based surveillance network with systematic testing for a panel of 17 RVs in pediatric and adult patients admitted for ARI has been in place since 2012-2013 during periods with high influenza circulation [27-30]. The same network was used for surveillance during the pandemic by adding SARS-CoV-2 to the panel and by extending surveillance periods to intense RV circulation. We report here the results of the comparison of RV contribution to hospitalization during 3 pandemic seasons (2020-21 to 2022-23) and 8 prepandemic seasons.

## Methods

### Population and Study Design

**Figure 1:**
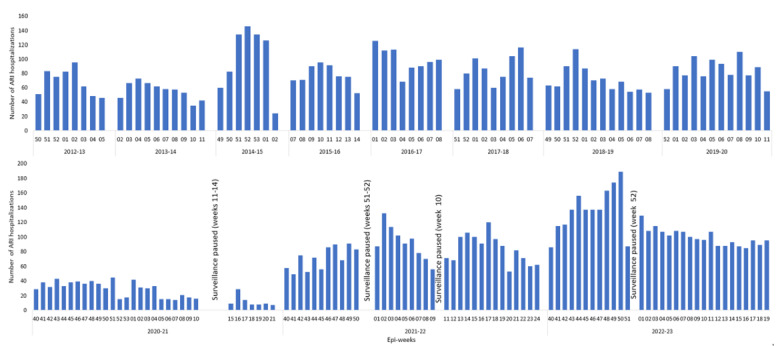
Weekly number of patients hospitalized for acute respiratory infection (ARI) included in active hospital-based prospective surveillance during the prepandemic (2012-20) and pandemic (2020-23) seasons in Québec, Canada. Four hospitals participated in the prepandemic period. Three hospitals participated in 2020-21 with periodic sampling by day of the week. Four hospitals participated in 2021-22 and 2022-23 with periodic sampling by day of the week. Two additional hospitals that joined in 2021-22 are not presented.

The characteristics of the Quebec prospective hospital-based surveillance network during the prepandemic years have been described in detail elsewhere [27-30]. In brief, 4 regional hospitals (2 community and 2 academic or tertiary; all of them serving both children and adults) with a catchment area of nearly 10% of the Quebec population (approximately 8.8 million in 2023) participated in the surveillance during 8 influenza seasons since 2012-13. One of the 4 hospitals (approximately 15% of the population included in previous years) was not able to participate in 2020-21 because of challenges with hospital resources during the pandemic, and it rejoined the network in 2021-22. Two additional tertiary hospitals, 1 adult and 1 pediatric hospital, joined the network in 2021-22, for a total of 6 hospitals (approximately 15% of the Quebec population). Results from these 2 hospitals are included only in the description of virus detection per week and are not used for comparison with the prepandemic period. All patients presenting to the emergency department with ARI were systematically swabbed during high influenza activity weeks of prepandemic years or during periods with increased hospitalizations due to SARS-CoV-2 or ORVs during the pandemic (Figure 1).

### Inclusion and Exclusion Criteria

Eligible patients were those who were admitted for ≥24 hours and who met a standardized ARI definition (fever or feverishness not attributed to another illness or cough or sore throat) that was expanded in 2020-21 to include symptoms specific for COVID-19 (adjusted from the Canadian Nosocomial Infection Surveillance Program [CNISP] [31]; fever or history of fever not attributed to another illness, or cough [or exacerbation of cough] or difficulty breathing [or exacerbation of difficulty breathing], or sudden extreme fatigue, or at least two of the following symptoms: rhinorrhea or nasal congestion, sore throat, myalgia or arthralgia, or sudden anosmia or ageusia). Nurses collected demographic and clinical details from the patient or legal representative on a standardized questionnaire and reviewed patients’ charts at discharge for additional clinical information. Patients with onset of ARI symptoms after admission, those who refused to consent or were unable to consent (during the period before the ethics committee exemption), those who did not meet the ARI definition, and those who were admitted for less than 24 hours were excluded from this analysis.

### Surveillance Period

**Figure 2:**
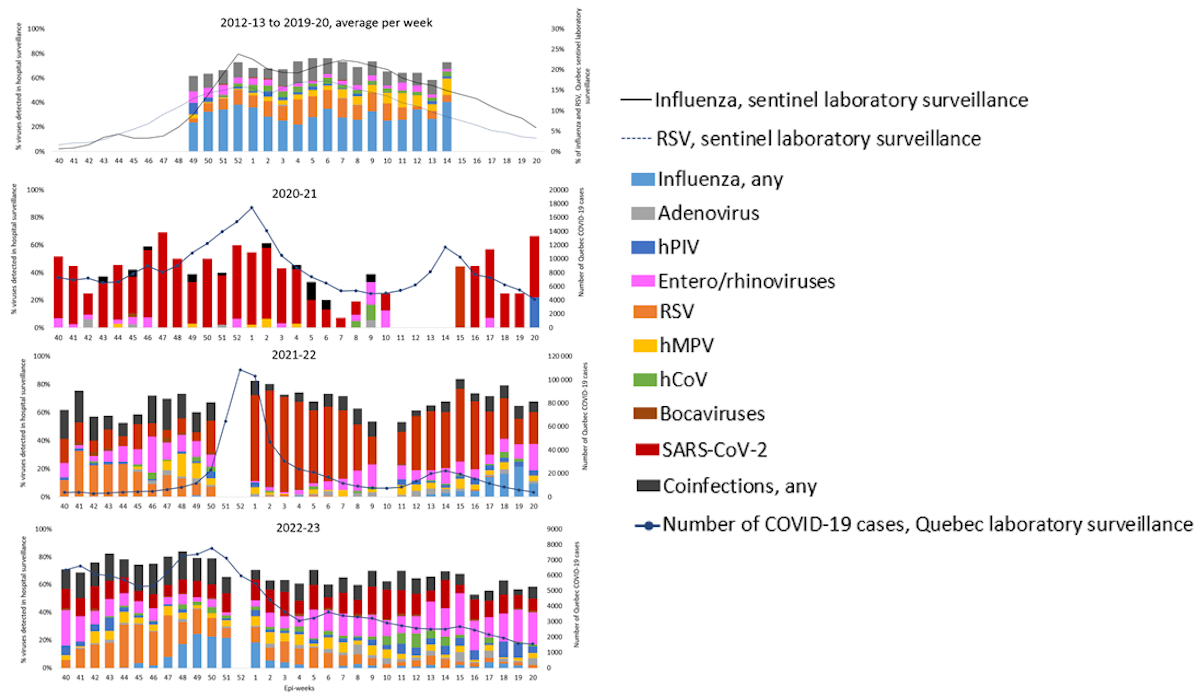
Proportion of respiratory virus detection among patients hospitalized for acute respiratory infection by epi-week in the hospital-based prospective surveillance network during the prepandemic (2012-20) and pandemic (2020-23) seasons in Québec, Canada. All participating hospitals are included (4 hospitals from 2012-13 to 2019-20, 3 hospitals in 2020-21, and 6 hospitals in 2021-22 and 2022-23). In order to simplify the presentation, epi-week 53 (2014-15 and 2019-20) is excluded. Weeks 40 to 20 are presented. The apparent increase in the proportion of hospitalizations due to influenza during epi-week 14 is due to the small number of hospitalized patients since only 1 season (2015-16) contributed. For consistency with other seasons, data for epi-weeks 40 to 20 during the pandemic seasons are presented, although the surveillance period was longer for some pandemic years. hCoV: common human coronavirus; hMPV: human metapneumovirus; hPIV: human parainfluenza virus; RSV: respiratory syncytial virus.

For the prepandemic years, the surveillance period started when the positivity rate for influenza in respiratory specimens from the provincial sentinel laboratory surveillance was ≥15% for 2 consecutive weeks and stopped the week after this rate dropped below 15% or when the planned sample size for the season was achieved (800-1000 specimens depending on the season). The provincial laboratory surveillance included >40 laboratories across the province of Quebec with >100,000 respiratory specimens per year. Surveillance lasted from 7 to 12 weeks per season (median of 8.5 weeks) between epi-weeks 49 (earliest) and 14 (latest) (Figure 1). In 2020-21, the surveillance period (September 27, 2020 [epi-week 40] to May 29, 2021 [epi-week 21]; overall duration of 35 weeks) coincided with Québec’s second and third COVID-19 waves (caused by ancestral and Alpha SARS-CoV-2 variants) [32] (Figure 2; Multimedia Appendix 1). In 2021-22, the surveillance period (October 04, 2021 [epi-week 40] to June 18, 2022 [epi-week 24]; duration of 34 weeks) captured an unexpected interseasonal RSV surge along with the descending and ascending phases of the fourth (Delta variant), fifth (Omicron BA.1 variant), and sixth (Omicron BA.2 variant) waves. The 2 additional hospitals joined the surveillance in 2021, with the adult hospital starting at epi-week 43 and the pediatric hospital starting at epi-week 46. During 2022-23, the surveillance period lasted 32 epi-weeks (from October 02, 2022 [epi-week 40] to May 20, 2023 [epi-week 20]). Details of the epidemiologic situation in Quebec are presented in Multimedia Appendix 1.

Because of challenges with hospital resources during periods of high SARS-CoV-2 circulation, the surveillance was paused during some weeks and sampling of enrollment during predetermined days of the week was adopted by some hospitals (Figure 1).

### Laboratory Analysis

Nasal specimens collected on flocked swabs from eligible patients were sent to the provincial public health laboratory (Laboratoire de Santé Publique du Québec [LSPQ]) and tested using the Luminex NxTAG Respiratory Pathogen Panel assay that detects influenza A (subtypes H3 and H1); influenza B; respiratory syncytial virus (RSV) (A and B differentiated starting in 2016-17); human parainfluenza viruses (hPIVs) 1, 2, 3, and 4; human metapneumovirus (hMPV); common human coronaviruses (hCoVs) NL63, HKU1, 229E, and OC43; enteroviruses/rhinoviruses (not differentiated); adenovirus; bocavirus; and 3 bacteria (*Mycoplasma pneumoniae*, *Chlamydia pneumoniae*, and *Legionella pneumophila*). Nucleic acids were purified using the bioMerieux eMAG platform, and polymerase chain reaction (PCR) products were analyzed on a Luminex Magpix system, as prescribed by the manufacturer. NxTAG assays were approved for diagnosis by Health Canada.

This assay was systematically used during all prepandemic years and during all pandemic years in the hospitals included in the main analysis (comparison between prepandemic and pandemic seasons). Additional assays used by hospitals and contributing only to descriptive results (Figure 2) were as follows: (1) BioFire Respiratory Panel 2.1 (RP2.1) used for ORV testing by local laboratories (considered in the descriptive analysis for patients for whom specimens were not available to be tested by Luminex NxTAG); (2) in-house multiplex reverse transcription PCR (MRVP) detecting influenza A and B; hPIVs 1, 2, and 3; adenovirus; rhinovirus; enterovirus; hCoVs 229E and OC43; RSV; and hMPV (used by the adult center added to the surveillance starting at epi-week 43 in 2021) [33]; and (3) in-house PCR using LightMix Modular Assays according to the manufacturer’s recommendations [34] to detect influenza A and B, RSV, hCoV (not differentiated), hMPV, adenovirus, hPIV (not differentiated), enteroviruses/rhinoviruses (not differentiated), and SARS-CoV-2 (used by the pediatric center added to the surveillance starting at epi-week 46 in 2021). Throughout the pandemic years, SARS-CoV-2 was detected at local laboratories by using commercially available diagnostic tests.

### Statistical Analysis

Proportions were compared by using the chi-square or Fisher exact test when appropriate. Mean values were compared by using the Wilcoxon test. The Cochran-Armitage trend test was used to assess the linear trend of proportions across age categories. Statistical significance was set at *P*<.05. Statistical analyses were conducted using SAS version 9.4 (SAS Institute). A similar hospitalization rate and viral etiology distribution was assumed for days with and without enrollment during weeks with only 3 enrollment days.

### Ethical Considerations

Institutional Review Board approval was obtained from all participating hospitals (Hôpital régional de Rimouski [number: CCER 11-12-13], Hôpital de Chicoutimi [number: 2011-032], Hôpital de la Cité-de-la-Santé – Laval [number: 06.02.02/2011-2012], and Centre hospitalier universitaire régional de Trois-Rivières [number: 2011-016-00]) for the first 3 years, and a signed informed consent form, including the possibility of a secondary analysis, was used. A waiver was obtained for the following years when the project was conducted as sentinel surveillance mandated by the Ministry of Health from the Research Ethics Board of the Centre hospitalier universitaire de Québec-Université Laval (2019-4455), and it was considered exempt from the requirement for ethics approval. During pandemic years, surveillance was performed within the legal mandate of the National Director of Public Health of Quebec under the Public Health Act and did not require research ethics committee review. This retrospective analysis used deidentified data. No compensation was provided to patients.

## Results

### Surveillance Participants

Overall, 15,199 patients potentially eligible for surveillance were approached (6412 during the prepandemic period, 1454 in 2020-21, 3124 in 2021-22, and 4209 in 2022-23) (Multimedia Appendix 2). Patients missed by nurses (n=25) or those with samples not received by LSPQ or samples of insufficient volume (n=1213) were comparable to those included in the main analysis with respect to age (mean age 61 vs 62 years; *P*=.97) and sex (47% vs 49% female; *P*=.17). A total of 10,550 patients hospitalized for community-acquired ARI were included in the analysis: 5832 (1493 children aged 0-17 years and 4339 adults) during the 8 prepandemic influenza seasons, 791 (29 children and 762 adults) during the 2020-21 season, 1606 (341 children and 1265 adults) during the 2021-22 season, and 1894 (406 children and 1488 adults) during the 2022-23 season (Table 1).

**Figure 3:**
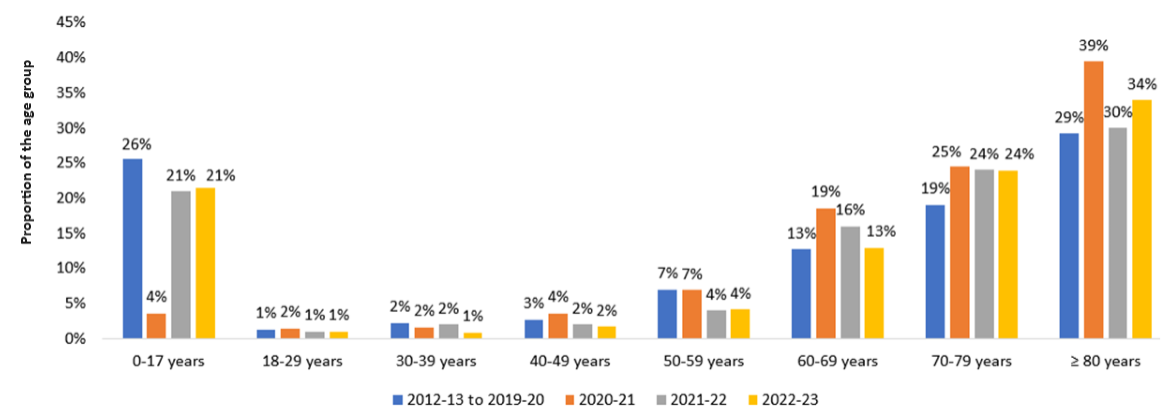
Age distribution of patients hospitalized for acute respiratory infection in the hospital-based prospective surveillance network in the prepandemic period (peaks of influenza seasons from 2012-13 to 2019-20) and 3 pandemic years (2020-21, 2021-22, and 2022-23) in Quebec, Canada.

When comparing the age distribution of hospitalized patients from the hospitals that participated in the surveillance since the beginning, the proportion of children was significantly lower in 2020-21 compared to prepandemic seasons (29/791, 3.7% vs 1493/5832, 25.6%; *P*<.001), and it increased and almost reached the levels observed during prepandemic seasons in the following 2 pandemic years (around 21% in both the second [341/1606, 21.2%] and third [406/1894, 21.4%] pandemic years; *P*<.001). During both prepandemic and pandemic seasons, the proportion of young adults among patients hospitalized with ARI was very low (1%-2% for those aged 18-29 years and those aged 30-39 years), and there was a subsequent gradual increase with age to 2%-4% in those aged 40-49 years, 4%-7% in those aged 50-59 years, 13%-19% in those aged 60-69 years, 19%-25% in those aged 70-79 years, and 29%-39% in those aged ≥80 years (*P*<.001 for the increase in the proportion for both prepandemic and pandemic seasons) (Figure 3). Compared to the prepandemic period, the proportion of patients aged ≥60 years was significantly higher during the first pandemic year (653/791, 82.6% vs 3561/5832, 61.1%; *P*<.001) and then decreased to varying degrees during the subsequent years (approximately 70% in 2021-22 [1123/1606, 69.9%] and 2022-23 [1341/1894, 70.8%]) but remained higher than that during the prepandemic period (*P*<.001), mirroring the decrease in pediatric hospitalizations but likely also associated with age-dependent SARS-CoV-2 severity (Figure 3).

**Table 1:** Number and proportion of patients hospitalized for acute respiratory infection by age group and detected respiratory virus in Quebec, Canada, during prepandemic (2012-20) and pandemic (2020-23) seasons.

Age group (years)	2012-20 (prepandemic, 4 hospitals)			2020-21 (pandemic, 3 hospitals)					2021-22 (pandemic, 4 hospitals)					2022-23 (pandemic, 4 hospitals)			
	Tested, n (%)	≥1 virus positive, n (%)	Tested, n (%)		≥1 virus positive, n (%)	SARS-CoV-2, with or without another virus, n (%)	Other virus without SARS-CoV-2, n (%)	Tested, n (%)		≥1 virus positive, n (%)	SARS-CoV-2, with or without another virus, n (%)	Other virus without SARS-CoV-2, n (%)	Tested, n (%)		≥1 virus positive, n (%)	SARS-CoV-2, with or without another virus, n (%)	Other virus without SARS-CoV-2, n (%)
0-17	1493 (25.6)	1384 (92.7)	29 (3.7)		17 (58.6)^a,b^	0 (0)	17 (58.6)	341 (21.2)		308 (90.3)^b^	30 (8.8)	278 (81.5)	406 (21.4)		361 (88.9)^a,b^	22 (5.4)	339 (83.5)
18-29	79 (1.4)	50 (63.3)	12 (1.5)		3 (25.0)^a^	2 (16.7)	1 (8.3)	10 (0.6)		6 (60.0)	4 (40.0)	2 (20.0)	19 (1.0)		11 (57.9)	4 (21.1)	7 (36.8)
30-39	130 (2.2)	75 (57.7)	13 (1.6)		6 (46.2)	6 (46.2)	0 (0)	26 (1.6)		16 (61.5)	12 (46.2)	4 (15.4)	17 (0.9)		10 (58.8)	3 (17.6)	7 (41.2)
40-49	160 (2.7)	104 (65.0)	29 (3.7)		16 (55.2)	16 (55.2)	0 (0)	35 (2.2)		23 (65.7)	19 (54.3)	4 (11.4)	32 (1.7)		21 (65.6)	8 (25.0)	13 (40.6)
50-59	409 (7.0)	267 (65.3)	55 (7.0)		28 (50.9)	26 (47.3)	2 (3.6)	71 (4.4)		50 (70.4)^b^	38 (53.5)	12 (16.9)	79 (4.2)		36 (45.6)^a^	12 (15.2)	24 (30.4)
60-69	744 (12.8)	452 (60.8)	147 (18.6)		48 (32.7)^a,b^	44 (29.9)	4 (2.7)	260 (16.2)		128 (49.2)^a,b^	92 (35.4)	36 (13.8)	245 (12.9)		103 (42.0)^a^	40 (16.3)	63 (25.7)
70-79	1111 (19.1)	678 (61.0)	194 (24.5)		78 (40.2)^a,b^	73 (37.6)	5 (2.6)	382 (23.8)		202 (52.9)^a,b^	146 (38.2)	56 (14.7)	453 (23.9)		201 (44.4)^a^	92 (20.3)	109 (24.1)
≥80	1706 (29.3)	1097 (64.3)	312 (39.4)		154 (49.4)^a,b^	140 (44.9)	14 (4.5)	481 (30.0)		306 (63.6)^b^	241 (50.1)	65 (13.5)	643 (33.9)		364 (56.6)^a,b^	178 (27.7)	186 (28.9)
≥60	3561 (61.1)	2227 (62.5)	653 (82.6)		280 (42.9)^a^	257 (39.4)	23 (3.5)	1123 (69.9)		636 (56.6)^a,b^	479 (42.7)	157 (14.0)	1341 (70.8)		668 (49.8)^a,b^	310 (23.1)	358 (26.7)
Total	5832 (100)	4107 (70.4)	791 (100)		350 (44.2)^a,b^	307 (38.8)	43 (5.4)	1606 (100)		1039 (64.7)^a,b^	582 (36.2)	457 (28.5)	1894 (100)		1107 (58.4)^a,b^	359 (19.0)	748 (39.5)

^a^*P*<.05 for comparison with 2012-20; Fisher exact test.

^b^*P*<.05 for comparison between the first pandemic year (2020-21) and each of the subsequent years (2021-22 and 2022-23).

### Viral Etiology

#### Overall Hospitalizations

During the prepandemic seasons, at least one respiratory virus was detected in 70.4% (4107/5832) of the patients hospitalized for ARI (Table 1). The most frequently detected viruses across all age groups were influenza (2107/5832, 36.1%), RSV (1080/5832, 18.5%), hMPV (475/5832, 8.1%), enteroviruses/rhinoviruses (460/5832, 7.9%), and hCoV (376/5832, 6.4%). Coinfections were detected in 12.3% (720/5832) of patients.

In 2020-21, the most frequently detected virus was SARS-CoV-2 (307/791, 38.8%). ORVs were detected in 5.4% (43/791) of patients, and the most frequent were enteroviruses/rhinoviruses (26/791, 3.3%), hMPV (11/791, 1.4%), and adenoviruses (10/791, 1.3%). No influenza or RSV was detected. Coinfections were detected in 1.8% (14/791) of patients: 1.0% (8/791) involved a combination of ORVs without SARS-CoV-2 and 0.8% (6/791) involved SARS-CoV-2 with ORVs.

In 2021-22, the most frequently detected viruses were SARS-CoV-2 (582/1606, 36.2%), RSV (162/1606, 10.1%), enteroviruses/rhinoviruses (153/1606, 9.5%), influenza viruses (61/1606, 3.8%; all being influenza A [H3N2]), adenoviruses (59/1606, 3.7%), and bocaviruses (55/1606, 3.4%). ORVs (excluding those with SARS-CoV-2 coinfection) were detected in 28.5% (457/1606) of patients. Coinfections were detected in 9.5% (152/1606) of patients: 7.3% (118/1606) involved a combination of ORVs without SARS-CoV-2 and 2.1% (34/1606) involved SARS-CoV-2 with ORVs. The peak of hospitalization due to RSV was detected 16 weeks earlier (epi-week 40) compared to prepandemic seasons (on average, epi-week 4) (Figure 2). The peak of hospitalization due to influenza occurred 17 weeks later in 2021-22 (epi-week 17) than the average peak at epi-week 52 during prepandemic seasons (Figure 2).

In 2022-23, the most frequently detected viruses were SARS-CoV-2 (359/1894, 19.0%), RSV (215/1894, 11.4%), influenza viruses (134/1894, 7.1%; mostly influenza A [H3N2] at 88.8% [119/134]), and enteroviruses/rhinoviruses (213/1894, 11.2%). ORVs without SARS-CoV-2 were detected in 39.5% (748/1894) of patients. Coinfections were detected in 8.7% (164/1894) of patients: 6.9% (130/1894) involved a combination of ORVs without SARS-CoV-2 and 1.8% (34/1894) involved SARS-CoV-2 with ORVs. The timing of influenza and RSV hospitalization was more aligned with historical time frames, although differences still occurred (influenza peaked 4 weeks earlier [epi-week 48 vs epi-week 52]; RSV peaked 15 weeks earlier [epi-week 41 vs epi-week 4]) (Figure 2).

#### Pediatric Hospitalizations

During the prepandemic period, at least one RV was detected in 92.7% (1384/1493) of children, including 30.5% (456/1493) coinfections. Pediatric hospitalizations due to RVs sharply decreased during the first pandemic year. The detection rate decreased to 58.6% (17/29) in 2020-21 (7/29, 24.1% being coinfections) and reverted to a rate comparable to that in the prepandemic period in 2021-22 (308/341, 90.3%) and 2022-23 (361/406, 88.9%). A similar trend was observed for coinfections, with a decrease to 24.1% (7/29) in 2020-21 and an increase to a rate closer to that in the prepandemic years during the 2 subsequent years (110/341, 32.3% in 2021-22 and 128/406, 31.5% in 2022-23).

Prior to the COVID-19 pandemic, the most frequently detected viruses in hospitalized children were RSV (721/1493, 48.3%) and influenza (365/1493, 24.4%) (Table 2). In 2020-21, SARS-CoV-2, RSV, and influenza were not detected in any hospitalized child, and hospitalizations with an identified virus in children were due to enteroviruses/rhinoviruses (8/29, 27.6%), adenoviruses (7/29, 24.1%), and bocaviruses (6/29, 20.7%). The proportions of detected adenoviruses and bocaviruses were significantly higher than those reported during the prepandemic seasons (Table 2), and no significant differences were detected for the rest of the viruses. In the 2 subsequent pandemic years, RSV was again the most frequently detected virus in children, while influenza and SARS-CoV-2 had a low etiological contribution to pediatric ARI hospitalizations. In 2021-22, most of the hospitalizations in children were due to ORVs without SARS-CoV-2 (278/341, 81.5%), and only 8.8% (30/341) were due to SARS-CoV-2 (including 10 coinfections with ORVs). The predominant virus was RSV (115/341, 33.7%), followed by enteroviruses/rhinoviruses (110/341, 32.3%) and bocaviruses (49/341, 14.4%). Compared to prepandemic seasons, the contribution was significantly higher for enteroviruses/rhinoviruses, hPIV 1-4, and bocaviruses, and lower for RSV, influenza, and hCoV (Table 2). In 2022-23, most of the hospitalizations were due to ORVs (339/406, 83.5%), and 5.4% (22/406) were due to SARS-CoV-2 (including 13 coinfections with ORVs). The predominant virus was again RSV (158/406, 38.9%), followed by enteroviruses/rhinoviruses (114/406, 28.1%), bocaviruses (53/406, 13.1%), hMPV (39/406, 9.6%), hCoV (38/406, 9.4%), adenoviruses (35/406, 8.6%), influenza (29/406, 7.1%), and hPIV (26/406, 6.4%). Compared to prepandemic seasons, significantly less cases of RSV and influenza and more cases of enteroviruses/rhinoviruses, bocaviruses, and hPIV were detected.

Some differences in the age distribution of children hospitalized before or during the pandemic were observed regarding some etiological viruses. For example, the average age among children hospitalized with RSV was 16 months in 2022-23, which was higher than in the prepandemic period (8 months) and in 2021-22 (7 months; *P*<.001). Differences in the average age of children hospitalized with enteroviruses/rhinoviruses were also observed but to a lower degree. It was higher in 2022-23 (21 months) and 2021-22 (20 months) than in the prepandemic period (17 months; *P*<.001).

**Table 2:** Children hospitalized for acute respiratory infection and detected viruses in Quebec, Canada, during prepandemic (2012-20) and pandemic (2020-23) seasons.

Variable						Peak of the 2012-20 influenza season (4 hospitals)					2020-21 (3 hospitals)		2021-22 (4 hospitals)		2022-23 (4 hospitals)
						Yearly average number (minimum-maximum)			Detection rate (N=1493), n (%)	Detection rate (N=29), n (%)		Detection rate (N=341), n (%)		Detection rate (N=406), n (%)	
Number of included patients						187 (113-322)			N/A^a^	N/A		N/A		N/A	
**At least one respiratory virus (not mutually exclusive)**						173 (103-307)			1384 (92.7)	17 (58.9)^b^		308 (90.3)		361 (88.9)^b^	
	**Influenza, any**				45.5 (23-96)			365 (24.4)			0 (0)^b^		24 (7.0)^b^		29 (7.1)^b^
		**Influenza A**		34.3 (22-57)			274 (18.4)			0 (0)		24 (7.0)^b^		29 (7.1)^b^	
			H3N2	15.3 (0-32)			122 (8.2)			0 (0)		24 (7.0)		25 (6.2)	
			H1N1	18.5 (0-49)			148 (9.9)			0 (0)		0 (0)^b^		3 (0.7)^b^	
			A unsubtyped	0.5 (0-2)			4 (0.3)			0 (0)		0 (0)		1 (0.2)	
		Influenza B		11.4 (0-61)			91 (6.1)			0 (0)		0 (0)^b^		0 (0)^b^	
	RSV^c^				90.1 (44-172)			721 (48.3)			0 (0)^b^		115 (33.7)^b^		158 (38.9)^b^
	Adenovirus				10.9 (4-20)			87 (5.8)			7 (24.1)^b^		28 (8.2)		35 (8.6)
	hMPV^d^				20.4 (0-38)			163 (10.9)			0 (0)		40 (11.7)		39 (9.6)
	hPIV^e^ 1-4				7.3 (0-13)			58 (3.9)			0 (0)		30 (8.8)^b^		26 (6.4)^b^
	hCoV^f^				19.3 (7-37)			154 (10.3)			3 (10.3)		14 (4.1)^b^		38 (9.4)
	Enteroviruses/rhinoviruses				30.0 (22-48)			240 (16.1)			8 (27.6)		110 (32.3)^b^		114 (28.1)^b^
	Bocaviruses				16.9 (6-33)			135 (9.0)			6 (20.7)^b^		49 (14.4)^b^		53 (13.1)^b^
	SARS-CoV-2				N/A			N/A			0 (0)		30 (8.8)		22 (5.4)
**Respiratory viruses without SARS-CoV-2**															
	Monoinfection				116 (69-231)			928 (62.2)			10 (34.5)		178 (52.2)		224 (55.2)
	Coinfection, any RV^g^ without SARS-CoV-2				57 (27-91)			456 (30.5)			7 (24.1)		100 (29.3)		115 (28.3)
**SARS-CoV-2**															
	Monoinfection				N/A			N/A			0 (0)		20 (5.9)		9 (2.2)
	Coinfection, SARS-CoV-2 + any RV				N/A			N/A			0 (0)		10 (2.9)^h^		13 (3.2)^i^
Total coinfections						57 (27-91)			456 (30.5)	7 (24.1)		110 (32.2)		128 (31.5)	

^a^N/A: not applicable.

^b^*P*<.05 for comparison with 2012-20; Fisher exact test.

^c^RSV: respiratory syncytial virus.

^d^hMPV: human metapneumovirus.

^e^hPIV: human parainfluenza virus.

^f^hCoV: common human coronavirus.

^g^RV: respiratory virus.

^h^SARS-CoV-2 in coinfection with adenoviruses (n=4), hMPV (n=2), and enteroviruses/rhinoviruses (n=2).

^i^SARS-CoV-2 in coinfection with RSV (n=4), adenoviruses (n=3), bocaviruses (n=2), hCoV (n=2), hMPV (n=1), and enteroviruses/rhinoviruses (n=1).

#### Adult Hospitalizations

During the prepandemic seasons, at least one RV was detected in 62.8% (2723/4339) of adults (264/4339, 6.1% coinfections), and influenza was the most predominant virus (1742/4339, 40.1% globally and 1742/2723, 63.9% of all detected viruses).

During all pandemic years, significantly fewer adults (1810/3515, 51.5%) were positive for at least one RV (including SARS-CoV-2 and ORVs) in comparison to the prepandemic period (2723/4339, 62.8%). With a few exceptions, the detection rate was comparable to prepandemic years in the younger age groups (18-59 years) and was lower in those aged ≥60 years (Table 1). SARS-CoV-2 was the most frequently identified virus during all 3 pandemic years among adults hospitalized for ARI (2020-21: 307/762, 40.3%; 2021-22: 552/1265, 43.6%; and 2022-23: 337/1488, 22.6%) (Tables 1 and 3). At least one ORV was detected in 3.4% (26/762), 14.2% (179/1265), and 27.5% (409/1488) of adults in 2020-21, 2021-22, and 2022-23, respectively. Except for enteroviruses/rhinoviruses and hPIV in 2022-23, all individual viruses were detected in lower proportions during the pandemic period compared to the prepandemic period, although differences did not always reach statistical significance owing to low numbers (Table 3). 

A sensitivity analysis, which excluded comparisons of the hospital that did not participate during the first pandemic year, did not reveal differences in detected trends (data not presented).

The results of viral detection in the 2 additional hospitals in 2021-22 and 2022-23 compared to the 4 hospitals included in the main analysis are presented in Multimedia Appendix 3. Differences might be explained by the different assays used for laboratory analyses, the populations (only pediatric patients in one hospital and only adults in the other hospital), and the time spans. While comparisons with prepandemic seasons are not appropriate in this context, we included results from the additional hospitals in the presentation of viral etiology by week in order to illustrate a more comprehensive impact of RVs on hospitalization (Figure 2).

**Table 3:** Adults hospitalized for acute respiratory infection and detected viruses in Quebec, Canada, during prepandemic (2012-20) and pandemic (2020-23) seasons.

Variable						Peak of the 2012-20 influenza season (4 hospitals)							2020-21 (3 hospitals)				2021-22 (4 hospitals)				2022-23 (4 hospitals)
						Yearly average number (minimum-maximum)			Detection rate (N=4339), n (%)			Detection rate (N=762), n (%)				Detection rate (N=1265), n (%)				Detection rate (N=1488), n (%)	
Number of included patients						542.8 (388-689)			N/A^a^			N/A				N/A				N/A	
**At least one respiratory virus (not mutually exclusive)**						340.4 (224-425)			2723 (62.8)			333 (43.7)^b^				731 (57.8)^b^				746 (50.1)^b^	
	**Influenza, any**				217.1 (132-334)			1742 (40.1)			0 (0)^b^				37 (2.9)^b^				105 (7)^b^		
		**Influenza A**			185.4 (109-324)			1483 (34.2)			0 (0)^b^				37 (2.9)^b^				105 (7)^b^		
			H3N2	125.8 (2-316)			1006 (23.2)			0 (0)^b^				37 (2.9)^b^				94 (6)^b^			
			H1N1	57.0 (0-123)			456 (10.5)			0 (0)^b^				0 (0)^b^				11 (0.7)^b^			
			A unsubtyped	2.6 (0-8)			21 (0.5)			0 (0)				0 (0)^b^				0 (0)^b^			
		Influenza B			32.4 (1-146)			259 (6.0)			0 (0)^b^				0 (0)^b^				0 (0)^b^		
	RSV^c^				44.9 (19-75)			359 (8.3)			0 (0)^b^				47 (3.7)^b^				57 (3.8)^b^		
	Adenovirus				1.6 (0-5)			13 (0.3)			3 (0.4)				31 (2.5)^b^				7 (0.5)		
	hMPV^d^				39.0 (2-103)			312 (7.2)			11 (1.4)^b^				20 (1.6)^b^				83 (6)^b^		
	hPIV^e^ 1-4				14.5 (3-25)			116 (2.7)			0 (0)^b^				18 (1.4)^b^				56 (3.8)^b^		
	hCoV^f^				27.8 (7-49)			222 (5.1)			0 (0)^b^				17 (1.3)^b^				37 (2.5)^b^		
	Enteroviruses/rhinoviruses				27.5 (12-43)			220 (5.1)			18 (2.4)^b^				43 (3.4)^b^				99 (7)^b^		
	Bocaviruses				2.5 (0-6)			20 (0.5)			2 (0.3)				6 (0.5)				2 (0.1)		
	SARS-CoV-2				N/A			N/A			307 (40.3)				552 (43.6)				337 (22.6)		
**Respiratory viruses without SARS-CoV-2**																					
	Monoinfection				307.4 (231-389)			2459 (56.7)			25 (3.2)				161 (12.7)				394 (26.5)^b^		
	Coinfection, any RV^g^ without SARS-CoV-2				33 (12-76)			264 (6.1)			1 (0.1)				18 (1.4)				15 (1.0)^b^		
**SARS-CoV-2**																					
	Monoinfection				N/A			N/A			301 (39.5)				528 (41.7)				316 (21.2)		
	Coinfection, SARS-CoV-2 + any RV				N/A			N/A			6 (0.8)^h^				24 (1.9)^i^				21 (1.4)^j^		
Total coinfections						33 (12-76)			264 (6.1)			7 (0.9)				42 (3.3)				36 (2.4)	

^a^N/A: not applicable.

^b^*P*<.05 for comparison with 2012-20; Fisher exact test.

^c^RSV: respiratory syncytial virus.

^d^hMPV: human metapneumovirus.

^e^hPIV: human parainfluenza virus.

^f^hCoV: common human coronavirus.

^g^RV: respiratory virus.

^h^SARS-CoV-2 in coinfection with hMPV (n=4), adenoviruses (n=2), enteroviruses/rhinoviruses (n=1), and bocaviruses (n=1).

^i^SARS-CoV-2 in coinfection with adenoviruses (n=13), hPIV (n=1), hCoV (n=3), enteroviruses/rhinoviruses (n=4), influenza A (H3N2; n=3), and RSV (n=1).

^j^SARS-CoV-2 in coinfection with hCoV (n=6), hMPV (n=5), enteroviruses/rhinoviruses (n=3), adenoviruses (n=2), influenza A (H3N2; n=2), hPIV (n=2), and RSV (n=1).

## Discussion

### Principal Findings

Our report, based on the systematic detection of RVs, describes changes in the etiology of pediatric and adult ARI hospitalizations during 3 pandemic years compared with 8 prepandemic winter seasons in the same population. To our knowledge, this is the longest time span of follow-up during both pandemic and prepandemic periods in both pediatric and adult hospitalized patients. We detected continuing changes in viral etiology and age distribution during the 3 pandemic years compared to the prepandemic period. While SARS-CoV-2 was the most frequent viral etiology of ARI hospitalizations during the 3 pandemic years, it was absent among children during the first year and scarcely detected during the second and third years. ORVs were the most important contributors to pediatric ARI hospitalization during all 3 pandemic years. In adults, SARS-CoV-2 was the most important contributor to ARI hospitalization during the first 2 pandemic years, but its relative importance gradually decreased, mirroring the increasing role of ORVs, and dropped below ORVs' detection during the third pandemic year. The most striking differences in age distribution were detected during the first pandemic year, mainly due to a remarkable decrease in pediatric ARI hospitalization.

### Interpretation of the Findings

The differences in the etiology and age distribution of ARI hospitalizations were a consequence of changes in both ORV and SARS-CoV-2 circulation and their impacts on hospitalizations, reflecting the intensity of mitigation measures and the resulting modification of immunity in the population following extended periods of the absence of some viruses (such as RSV). For instance, the circulation of ORVs was very low in 2020-21 when more stringent measures were implemented, increased during autumn 2021 following the easing of some measures, declined in December 2021 following the tightening of the measures in response to the Omicron wave, and increased again in February-March 2022 following broader travelling and school opening in Quebec (Multimedia Appendix 1) [35]. The increase in the average age of children hospitalized with RSV and enteroviruses/rhinoviruses during the pandemic period suggests that younger children had less exposure to these viruses due to the mitigation measures deployed in 2020-21. Another potential contributing factor was the evolution of SARS-CoV-2 variants. For example, during the 2020-21 season when ancestral and Alpha SARS-CoV-2 variants (affecting mostly adults) circulated, the pediatric population was spared, while during the autumn and winter of 2021-22 when Delta and then Omicron variants predominated [32], the pediatric population was more affected. Finally, COVID-19 vaccine uptake and effectiveness, and outpatient antiviral availability, which had an impact on preventing hospitalizations, varied by age group. During the second pandemic year, older patients were prioritized in the COVID-19 vaccination campaigns, and more patients with comorbidities were offered outpatient antiviral treatment. For children, however, COVID-19 vaccines were available later, and in young adults, vaccine uptake was lower than in the older population [36]. Therefore, the impact on SARS-CoV-2 hospitalizations varied by age and time period. The effectiveness of COVID-19 vaccines and their impact on the SARS-CoV-2 portion of ARI hospitalizations varied depending on the type of vaccine, the frequency of doses and delays between dose administrations, and the proportion of the population with prior COVID-19 infection, which steadily increased with the unfolding of the pandemic, allowing the development of hybrid immunity [37].

Overall, during the prepandemic influenza seasons, the 2 most frequently detected viruses among patients hospitalized with ARI were influenza and RSV. SARS-CoV-2 was the most important respiratory virus during the first pandemic year. In 2021-22 and 2022-23, its contribution decreased, while the contribution of ORVs increased. However, there were differences between the pediatric and adult populations. RVs (mostly RSV) affected more children than adults during the prepandemic winter seasons. Although the overall impact of ORVs was lower during the pandemic period, children remained mostly affected by ORVs and not by SARS-CoV-2 during all pandemic years. In adults, influenza was the most important virus during the prepandemic years, whereas SARS-CoV-2 was more important than ORVs during the first 2 pandemic years, while their roles reversed during the third pandemic year. It is of note that with the addition of the contribution of SARS-CoV-2 to ARI hospitalizations, the relative role of ORVs decreased during the pandemic years compared to the prepandemic period both in children and adults [38,39], with some minor exceptions (eg, enteroviruses/rhinoviruses) [40,41].

Nonenveloped viruses (rhinoviruses and adenoviruses) were less impacted by preventive measures aiming to halt SARS-CoV-2 transmission [40-42]. Their detection has been reported in hospitalized patients during periods of decreased detection of other RVs [43,44]. In our study, among children, the proportion of adenoviruses and enteroviruses/rhinoviruses was higher during the first pandemic year compared to the prepandemic period, and the proportion of enteroviruses/rhinoviruses remained higher during the second and third pandemic years. Among adults, the proportion of enteroviruses/rhinoviruses was higher during the third pandemic year. Since our study did not include less severe infections not leading to hospitalization, our findings reflect not only the circulation of these viruses, but also their virulence that may vary by age and season. It is of note that the proportion of enteroviruses/rhinoviruses might have been underestimated in the prepandemic period of our study because it was conducted only during the peaks of influenza circulation, and the weeks of the most intense circulation of enteroviruses/rhinoviruses may not have been captured.

The important reduction of pediatric respiratory hospitalization, virtual absence of influenza and RSV during the first pandemic year, and increased role of COVID-19 and ORVs, especially RSV, in hospitalized children during the second and third pandemic years in our study are in line with reports from some other countries and other Canadian provinces [14,23,24,45,46]. Of note, the timing of the unusual surge of RSV varied by region of the world. In Europe, Israel, and the United States, increases were reported in the Spring-Summer of 2021 [14,23,24,45]. Major shifts in the epidemiology of RSV with large-scale outbreaks were reported in New Zealand (corresponding to the usual season) [47] and Australia (out of season) [16]. In Canada, the first province to report a surge of RSV was Quebec in August 2021, while the other provinces followed with a delay of 2 to 3 months [46]. In 2022, an earlier than usual start of the RSV season was reported in the Northern Hemisphere [17,21], with a peak of RSV-related hospitalizations between October and December [19,21]. The surge in RSV hospitalizations and the shift in the average age of children hospitalized with RSV (older in 2021-22) observed in our study are consistent with the results of other studies [48,49], and the findings might be attributable to the “immunity debt” that occurred because of the lack of exposure during the first pandemic year. However, it is not clear why important variability in the increase of the RSV hospitalization rate occurred globally, for example, it was relatively comparable to the prepandemic period in Germany but higher in the United States [19,21]. After a prolonged absence, the influenza season of 2021-22 occurred later than during prepandemic years in the Northern Hemisphere and affected mostly adults [20,50,51]. A shorter season than the typical prepandemic season was reported in Canada and Europe [51], while a longer season occurred in the United States [52]. During the third pandemic year (2022-23), an unusually early influenza season was reported in other Canadian provinces [20], Europe [53], and the United States [54], which is similar to our findings.

Evidence from across the world suggests that RV-specific seasonality is being progressively re-established [17,20,53,55]. However, it is yet unclear how the co-circulation of different viruses, now with the addition of SARS-CoV-2, will impact the occurrence and severity of ARI hospitalizations. Systematic testing for a panel of RVs allowed us to detect a high proportion of coinfection (>30%, including both SARS-CoV-2 and ORVs) in children during both the prepandemic and pandemic periods (with the exception of a somewhat lower proportion in the first pandemic year) and <10% coinfection in adults. However, our analysis did not aim to study the severity of coinfection or potential viral interference since we did not assess the expected coinfection rate, which requires additional epidemiological data. Viral interference between RVs (positive [additive or synergistic] or negative [antagonistic]) has been demonstrated at the cellular, host, and population levels before the pandemic [56]. SARS-CoV-2 could also interact with existing RVs. For example, it has been reported in a retrospective population-based cohort study that children with prior SARS-CoV-2 infection are more likely to have an RSV infection [57]. Moreover, rhinovirus infection may reduce the likelihood of SARS-CoV-2 infection according to in vitro assays [56].

### Study Limitations

This study had some limitations. First, the prepandemic surveillance occurred during the peaks of influenza activity, and therefore, the relative contribution of other RVs may be underestimated as compared to the entire winter season. Surveillance periods during the pandemic years were mostly tailored to the increase in respiratory hospitalizations following increased SARS-CoV-2 or ORV circulation and were much longer. However, we believe that the comparison is still valid because it includes periods with the most strain on hospital capacity due to intensive circulation of ORVs, SARS-CoV-2, or both. Second, the ARI definition used during the pandemic years was broader and less specific for some RVs than during the prepandemic years and may have contributed to lower detection of RVs. In addition, it may have contributed to an increase in older age groups in which symptoms, such as exacerbation of difficulty breathing and sudden extreme fatigue, may be associated with other nonrespiratory conditions. On the other hand, this broad definition may have decreased the probability of missing patients in whom RVs may have contributed to the deterioration of their condition. Third, the first pandemic year was limited to only 3 hospitals, and periodic pauses and sampling of enrollment during the 2 pandemic years were necessary given the stretched resources, which limited the sample size. However, this should not have influenced the relative contribution of RVs and global comparisons, as the surveillance period during pandemic years was much longer than prepandemic seasons and sensitivity analysis did not reveal differences in trends when excluding the hospital not included in the first pandemic year. In addition, this approach allowed the maintenance of surveillance in the context of resource challenges during the pandemic. Fourth, 19% of eligible patients hospitalized for ARI were excluded from the main analysis. However, we do not believe that this impacted the validity of comparisons made in this study given that age was the main factor driving differences in RV detection and there was no difference by age and gender in missed eligible patients compared to those included in the study. Finally, our results may not necessarily be extrapolated to other regions or periods of time because of temporal and geographical differences in ORVs and SARS-CoV-2 epidemiology.

### Study Strengths

The main strength of this study was the systematic testing for a broad panel of RVs for all admitted patients with symptoms of ARI during surveillance periods, by using the same diagnostic assay during all study periods. Moreover, it included both pediatric and adult populations during a total of 11 years, with the participation of at least three hospitals from different Quebec regions in each year, a broad case definition, a comprehensive detection of 17 RVs and SARS-CoV-2 potentially contributing to ARI hospitalizations, and a possibility to distinguish community-acquired infections from health care–acquired infections.

### Conclusion

Important shifts in viral etiology, seasonality, and age distribution of ARI hospitalizations in children and adults were observed during the 3 pandemic years compared to the prepandemic period. While the first pandemic season was significantly different from the prepandemic winter seasons, the second and third years were more comparable in terms of both RV contribution and age distribution. The complex interplay among mitigation measures, intrinsic seasonality and secular trends of ORVs, changes in circulating SARS-CoV-2 variants and their virulence, COVID-19 vaccine uptake and effectiveness, outpatient antiviral treatments, and potential viral interference may have played roles in the differential contribution of ORVs and SARS-CoV-2 to ARI hospitalizations. Our study underscores the importance of surveillance in understanding altered seasonal patterns of RVs and shows that the role of SARS-CoV-2 relative to ORVs is continually changing. The current situation may reflect a transition period until SARS-CoV-2 finds its ecological niche in the human population and ORVs re-establish their usual seasonal patterns. Although new SARS-CoV-2 variants may emerge and cause occasional increases in hospitalizations, in the long run, it may establish itself as another usual respiratory virus. At this point, it is difficult to foresee its role compared to ORVs; however, our study suggests that SARS-CoV-2 may continue to be the most important contributor to ARI hospitalizations in adults. Increased scrutiny of continuing changes in the etiology of ARI hospitalizations by using systematic multiplex testing approaches that allow valid comparisons, including assessment of observed and expected coinfections, is needed to inform mathematical modeling and appropriate public health recommendations.

## Appendix

Multimedia Appendix 1 Timeline of SARS-CoV-2 epidemiology and main mitigation measures in Quebec, Canada.

Multimedia Appendix 2 Surveillance flowchart by period in the hospitals participating in surveillance during the prepandemic (2012-2020) and pandemic (2020-2023) seasons in Québec, Canada.

Multimedia Appendix 3 Results of viral detection in patients hospitalized for acute respiratory infections in 2021-22 and 2022-23 (4 hospitals participating during the prepandemic period and 2 additional hospitals) in Québec, Canada.

## Abbreviations

ARI: acute respiratory infection
HCoV: common human coronavirus
hMPV: human metapneumovirus
hPIV: human parainfluenza virus
LSPQ: Laboratoire de Santé Publique du Québec
ORV: respiratory virus other than SARS-CoV-2
RSV: respiratory syncytial virus
RV: respiratory virus

## Data Availability

The data collected in this surveillance study are the property of the Quebec Ministry of Health (Ministère de la Santé et des Services sociaux du Québec) and have been shared with the research group under the legal mandate of the National Director of Public Health of Quebec under the Public Health Act precluding data sharing with a third party. Aggregate data are available within the manuscript and the multimedia appendix files. Real-time surveillance data from HospiVir are available online [58].
