# Supplementary material for: The Changing Landscape of Respiratory Viruses Contributing to Hospitalizations in Quebec, Canada: Results From an Active Hospital-Based Surveillance Study
Source: JMIR Public Health Surveill. 2024 May 6;10:e40792. doi: 10.2196/40792 (PMC11075779; doi:10.2196/40792)
Supplement: Multimedia Appendix 1 [file publichealth_v10i1e40792_app1.docx]

**Multimedia Appendix 1.** Timeline of SARS-CoV-2 epidemiology and main mitigation measures in Quebec, Canada.

| Year | Date | Events |
| --- | --- | --- |
| 2020 | **February 23** | **Start of first wave** |
|  | March 13 | Declaration of health emergency by the province of Quebec |
|  | March 14 | Cancellation of non-essential visits to long-term-care-facilities (LTCF) and hospitals; stay-at-home order for ≥70 years, physical distancing; closure of some public spaces, school and kindergartens closure |
|  | March 18 | International border closure |
|  | March 18 | LTCF and nursing homes lockdown, progressive closure of all services excepting those essential; restrictions of travel between some regions with check points at entry; cancellation of all summer festivals and activities |
|  | April 15 | Progressive opening of some sectors, easing of some measures |
|  | **July 11** | **End of first wave** |
|  | July 13 | Mandatory use of masks or face covering in public transport |
|  | July 18 | Mandatory use of masks or face covering in all public enclosed spaces |
|  | **August 20** | **Start of second wave** |
|  | September 8 | Graded regional warning system in place |
|  | September 11 | Progressive tightening of measures |
|  | December 14 | Start of COVID-19 vaccination with LTCF residents and LTCF health-care workers (HCW) according to priority order |
|  | December 25 | Closure of non-essential businesses |
|  | December 29 | First detection of the B.1.1.7 variant (Alpha) |
|  | December 31 | Given limited number of COVID-19 vaccines, dose 1 is prioritized in order to rapidly achieve higher coverage in vulnerable groups |
| 2021 | February 8 | Progressive easing of measures |
|  | February 9 | First detection of the B.1.351 variant (Beta) |
|  | March 1 | Vaccination extended to general population according to priority groups |
|  | **March 20** | **End of second wave** |
|  | **March 21** | **Start of third wave** |
|  | March 25 | Only one dose of vaccine may be administered to persons with prior COVID-19 infection |
|  | April 1 | Special emergency measures in some regions |
|  | April 26 | First detection of the B.1.617 variant (Delta) |
|  | May 18 | 50% of Quebec population ≥12 years received at least 1 dose |
|  | June 6 | 75% of Quebec population ≥12 years received at least 1 dose |
|  | **July 17** | **End of third wave** |
|  | **July 18** | **Start of forth wave** |
|  | September 1 | Vaccination passport required for access to most public spaces |
|  | September 28 | Booster dose recommended for LTCF and nursing homes residents |
|  | September 30 | 75% of Quebec population is adequately vaccinated |
|  | November 1 | 6 months between booster dose and last dose received is recommended. Optimal interval between 1^st^ and 2^nd^ dose is 8 weeks |
|  | November 15 | Progressive easing of some measures |
|  | November 16 | Booster dose recommended to ≥70 years from community, persons who received a viral vector vaccine may receive a mRNA vaccine booster |
|  | November 24 | Start of 5-11-year-olds vaccination |
|  | November 29 | Detection of omicron variant |
|  | December 4 | **End of forth wave** |
|  | December 5 | **Start of fifth wave** |
|  | December 20 | Progressive tightening of measures, including curfew starting December 31  Booster dose advanced for all ≥ 60 years; interval between last dose and booster shortened from 6 to 3 months |
|  | December 29 | Booster dose offered to essential workers followed-up by all population according to age groups |
| 2022 | January 5 | Change in PCR screening priorities and increased access to self-testing |
|  | January 17 | Curfew lifted |
|  | January 31 | Progressive easing of some of the measures |
|  | February 16 | Vaccination passports no longer required for some public spaces |
|  | February 18 | Booster dose offered to 12-17-year-olds (at least 3 months from the last dose) |
|  | February 21- March 12 | Further easing of measures |
|  | March 12 and 13 | **End of the fifth wave and the start of the sixth wave** |
|  | March 14 | The vaccine passport is no longer needed in most public places and health facilities |
|  | March 17 | Paxlovid^MC^ distributed in all pharmacies in Quebec. Access possible after a positive result (PCR or self-test). |
|  | March 29-May 5 | Booster dose (4^th^) recommended, in order of priority, to ≥80 years, ≥60 years, and ≥12 years from community |
|  | May 14 | Wearing masks in several enclosed or partially covered public places is no longer mandatory, except in public transportation and healthcare facilities. |
|  | May 28-29 | **End of sixth wave and the start of seventh wave** |
|  | June 18 | Lifting of the mask mandate in public transportation |
|  | July 21 | The Spikevax vaccine (Moderna) is authorized for children aged 6 months to 4 years |
|  | August 16 | Launch of the autumn vaccination campaign |
|  | September 3-4 | **End of the seventh wave and start of the high endemicity period^1^** |
|  | September 8 | Booster dose is available for those aged ≥5 years. |
|  | October 1 | Lifting COVID-19 border measures and travel requirements in Canada |
|  | November 16 | An advertising campaign is launched to encourage the vaccination against COVID-19 for those who received their last dose since ≥5 months or infected since ≥3 months |
| 2023 | April 6 | Easing of requirement to mask wearing in healthcare settings |
|  | August 26 | **End of the high endemicity period** |

^1-^active and sustained transmission at a relatively high level of SRAS-CoV-2 in the population over an extended period
