# Supplementary material for: The Changing Landscape of Respiratory Viruses Contributing to Hospitalizations in Quebec, Canada: Results From an Active Hospital-Based Surveillance Study
Source: JMIR Public Health Surveill. 2024 May 6;10:e40792. doi: 10.2196/40792 (PMC11075779; doi:10.2196/40792)
Supplement: Multimedia Appendix 2 [file publichealth_v10i1e40792_app2.docx]

**Multimedia Appendix 2.** Surveillance flowchart by period in the hospitals participating in surveillance during the prepandemic (2012-2020) and pandemic (2020-2023) seasons in Québec, Canada.

| Period | 2012-2020 (pre-pandemic, 4 hospitals) | 2020-2021  (first pandemic year, 3 hospitals) | 2021-2022 (second pandemic year, 4 hospitals) | 2022-2023 (third pandemic year, 4 hospitals) |
| --- | --- | --- | --- | --- |
| **Number of swabbed patients** | 6412 | 1454 | 3124 | 4209 |
| **Not eligible** |  |  |  |  |
| Refusal/unable to consent | 68 | NA | NA | NA |
| Definition not met^1^ | 51 | 63 | 689 | 937 |
| Enrolled before the surveillance period | 0 | 26 | 85 | 130 |
| Admitted less than 24 hours | 18 | 2 | 41 | 49 |
| COVID-19 patient transferred from another region^2^ | NA | 222 | NA | NA |
| Health-care acquired ARI^3^ | 364 | 145 | 120 | 253 |
| Other reason^4^ | 18 | 0 | 20 | 110 |
| Missed patients |  |  |  |  |
| Missed by nurses | 25 | NA | NA | NA |
| Samples not received by LSPQ/insufficient volume^5^ | 36 | 205 | 563 | 409 |
|  |  |  |  |  |
| **Included in the analysis** | 5832 | 791 | 1606 | 2321 |

NA: not applicable

^1^For example: symptoms related to another condition (i.e fever associated to urinary tract infection, cellulitis)

^2^Transfers from centers (located in another region not covered by participating hospitals) not designated to receive COVID-19 patients according to government mandate

^3^Defined as: Onset of ARI symptoms >72 hours after admission

^4^Duplicate cases.

^5^ Missed patients during pandemic years is explained by difficulty of specimen management during high volume of testing for SARS-CoV-2

Additional text

Distribution of reasons for non-eligibility differed between pre-pandemic (mostly patients with symptoms onset after admission, 64.8% (364/562 of those excluded)) and pandemic years (mostly definition not met, 41.5% (1689/4069) of those excluded). The most frequent reason for not meeting definition was a non-respiratory origin of fever or other non-specific symptoms (for example, urinary infection) which was not clear at the moment of enrolment. During the pre-pandemic period, 1% of the potentially eligible patients were either missed by nurses or their specimen was not tested by the central laboratory multiplex assay. During pandemic years, 13.4% (1177/8787) of hospitalized patients were missed because the sample was not received by central laboratory or received with insufficient volume. Higher proportion of missed patients during pandemic years in this category is explained by difficulty of specimen management during high volume of testing for SARS-CoV-2.
