# Supplementary material for: The Changing Landscape of Respiratory Viruses Contributing to Hospitalizations in Quebec, Canada: Results From an Active Hospital-Based Surveillance Study
Source: JMIR Public Health Surveill. 2024 May 6;10:e40792. doi: 10.2196/40792 (PMC11075779; doi:10.2196/40792)
Supplement: Multimedia Appendix 3 [file publichealth_v10i1e40792_app3.docx]

**Multimedia Appendix 3.** Results of viral detection in patients hospitalized for acute respiratory infections in 2021-22 and 2022-23 (4 hospitals participating during the prepandemic period and 2 additional hospitals) in Québec, Canada.

| Number of patients and type of infection | 2021-22 (4 main hospitals) | 2021-22 (two additional hospitals) | 2022-23 (4 main hospitals) | 2022-23 (two additional hospitals) |
| --- | --- | --- | --- | --- |
|  | Total number and detection rate, n(%) | Total number and detection rate, n(%) | Total number and detection rate, n(%) | Total number and detection rate, n(%) |
| Number of tested patients | 1606 | 1171 | 1894 | 1719 |
| At least one respiratory virus | 1039(64.7%) | 868(74.1%)^a^ | 1107 (58.4) | 1415 (82.3) ^a^ |
| Influenza, any | 61 (3.8%) | 49 (4.2%) | 134 (7.1) | 114 (6.6) |
| RSV | 162 (10.1%) | 36 (3.1%) ^a^ | 215 (11.4) | 458 (26.6) ^a^ |
| Adenovirus | 59 (3.7%) | 74 (6.3%) ^a^ | 42 (2.2) | 155 (9.0) ^a^ |
| hMPV | 60 (3.7%) | 78 (6.6%) ^a^ | 122 (6.4) | 131 (7.6) |
| hPIV 1-4 | 48 (3.0%) | 52 (4.4%) | 82 (4.3) | 109 (6.3) ^a^ |
| hCoV | 31 (1.9%) | 39 (3.3%) ^a^ | 75 (4.0) | 116 (6.7) ^a^ |
| Entero/rhinoviruses | 153 (9.5%) | 275 (23.5%) ^a^ | 213 (11.2) | 503 (29.3) ^a^ |
| Bocaviruses | 55 (3.4%) | 34 (2.9%) | 55 (2.9) | 36 (2.1) |
| SARS-CoV-2 | 582 (36.2%) | 383 (32.7%) | 359 (19.0) | 149 (8.7) ^a^ |
| Respiratory viruses without SARS-CoV-2 |  |  |  |  |
| Monoinfection | 339 (21.1%) | 394 (33.6%) ^a^ | 618 (32.6) | 982 (57.1) ^a^ |
| Co-infections, any RV without SARS-CoV-2 | 118(7.3%) | 91 (7.7%) | 130 (6.9) | 284 (16.5) ^a^ |
| SARS-CoV-2 |  |  |  |  |
| Monoinfection | 548 (34.1%) | 346 (29.5%) ^a^ | 325 (17.2) | 129 (7.5) ^a^ |
| Co-infections, SARS-CoV-2 + any RV | 34 (2.1%) | 37 (3.2%) | 34 (1.8) | 20 (1.2) |

RSV: respiratory syncytial virus; hMPV: human metapneumovirus, hPIV: human parainfluenza viruses 1, 2, 3, and 4, hCoV: common human coronaviruses; RV: respiratory viruses;

^a^ *P*<.05 for the comparison of additional hospital rates to the four main hospital for the same season.
